# New Methods for the Acoustic-Signal Segmentation of the Temporomandibular Joint

**DOI:** 10.3390/jcm11102706

**Published:** 2022-05-11

**Authors:** Marcin Kajor, Dariusz Kucharski, Justyna Grochala, Jolanta E. Loster

**Affiliations:** 1 Faculty of Electrical Engineering, Automatics, Computer Science and Biomedical Engineering, AGH University of Science and Technology, 30-059 Kraków, Poland; darekk@agh.edu.pl; 2 Department of Prosthodontics, Institute of Dentistry, Jagiellonian University Medical College, Jagiellonian University, 31-155 Kraków, Poland; justyna.lemejda@doctoral.uj.edu.pl (J.G.); jolanta.loster@uj.edu.pl (J.E.L.)

**Keywords:** temporomandibular joints, auscultation, segmentation, signal processing, deep learning

## Abstract

(1) Background: The stethoscope is one of the main accessory tools in the diagnosis of temporomandibular joint disorders (TMD). However, the clinical auscultation of the masticatory system still lacks computer-aided support, which would decrease the time needed for each diagnosis. This can be achieved with digital signal processing and classification algorithms. The segmentation of acoustic signals is usually the first step in many sound processing methodologies. We postulate that it is possible to implement the automatic segmentation of the acoustic signals of the temporomandibular joint (TMJ), which can contribute to the development of advanced TMD classification algorithms. (2) Methods: In this paper, we compare two different methods for the segmentation of TMJ sounds which are used in diagnosis of the masticatory system. The first method is based solely on digital signal processing (DSP) and includes filtering and envelope calculation. The second method takes advantage of a deep learning approach established on a U-Net neural network, combined with long short-term memory (LSTM) architecture. (3) Results: Both developed methods were validated against our own TMJ sound database created from the signals recorded with an electronic stethoscope during a clinical diagnostic trail of TMJ. The Dice score of the DSP method was 0.86 and the sensitivity was 0.91; for the deep learning approach, Dice score was 0.85 and there was a sensitivity of 0.98. (4) Conclusions: The presented results indicate that with the use of signal processing and deep learning, it is possible to automatically segment the TMJ sounds into sections of diagnostic value. Such methods can provide representative data for the development of TMD classification algorithms.

## 1. Introduction

Acoustic signals collected on the surface of the body carry diagnostic information and can be successfully used in the initial evaluation of various body parts, such as joints. In particular, auscultation is used in the diagnosis of the masticatory system. Conducted studies have shown that healthy temporomandibular joints (TMJ) work either noiselessly or generate only the expected physiological sounds. Thus, any unexpected noise in the TMJ acoustic signal can be used as an indicator of temporomandibular disorders (TMD) [1]. It is believed that the vast majority of TMDs are caused by the degeneration and perforation of the articular disc, which is especially prone to fatigue wear [2]. The most common symptoms of TMJ disorders are discomfort and pain, including limitations in the range of motion and the ability to open the jaw. Other symptoms that also decrease the quality of life include abnormal jaw movement, muscle tenderness, and bruxism.

According to studies, masticatory dysfunctions statistically affect up to 20% of the population [3,4]. TMJ disorders are a significant problem which often occur as a result of a stressful lifestyle. The pathogenesis of such dysfunctions may be directly related to daily parafunctions such as clenching and grinding [5]. Unfortunately, only 5% of people affected by disorders of the masticatory system undertake diagnostic procedures and treatment [6]. Judging by the cited research and statistical data, it is becoming increasingly appropriate to support TMD diagnosis with computational methods and automated classification algorithms.

**Figure 1:**
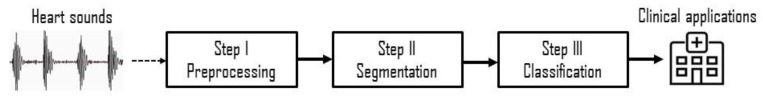
Generic pipeline for heart sounds classification [7].

One of the crucial steps of every biomedical signal-processing method is segmentation, which can be defined as the process of finding sections of diagnostic value in the repetitive signal. Figure 1 depicts the overall classification pipeline of heart sounds which can be generalized to other biomedical signals, including TMJ acoustic signals. Segmentation constitutes a separate step in the processing chain.

In the case of masticatory system auscultation, the patient is asked to periodically open and close the jaw to generate diagnostic segments representing the mandible movements. A phase between each representative movement is not relevant to the diagnosis and makes further signal processing less effective and prone to noise artifacts. Automated segmentation aims to extract only the abovementioned representative sections.

Based on the literature review, we found a lack of references concerning the segmentation of the TMJ auscultation signal. Phonocardiography, on the other hand, constitutes a field which has been studied in depth, and is well recognised in literature in the context of heart-sound segmentation. Therefore, we believe that it can become a reference for the processing and segmentation of sounds generated by other physiological systems, such as the masticatory system.

Sun et al. [8] obtained 97% accuracy in segmentation of the heart’s acoustic signal using the short-time modified Hilbert transform to calculate an envelope. This method was tested on 3390 s of heart sounds with diagnosed coronary artery disease, 3940 s with diagnosed rheumatic disease, 600 s without any diagnosed dysfunctions, and 1496.8 s of phonocardiographic signal from the Michigan Heart Sound and Murmur database (MHSDB). Varghees and Ramachandran [9] proved that Shannon entropy, in combination with the signal feature calculated on the basis of the instantaneous phase of the analytical signal, can be used for the effective segmentation of heart tones. The achieved mean sensitivity of the method was 99.43% and the positive predictive value (PPV) was 93.56%. The results were obtained using 701 signals, both noisy and clean, and physiological and pathological. Tang et al. [10] developed an algorithm for the segmentation of heart tones based on dynamic clustering built on the time-frequency parameters of a single heart cycle. They achieved an accuracy of 94.9% for the S1 tone (systolic) and 95.9% for the S2 tone (diastolic), which was tested on twenty-five people. Sedighian et al. [11] used implicit Markov models with homomorphic filtering and obtained an average accuracy of 92.4% for S1 segmentation and 93.5% for S2 segmentation by validation against the PASCAL database. Zhong et al. [12] presented a heart acoustic-signal-segmentation algorithm using the wavelet transform for low-pass filtering. The obtained outcomes were compared with the results achieved when applying the Hilbert transform. The wavelet transform turned out to be better for signals with a high noise level (88.29% sensitivity in comparison with 66.77% for the Hilbert method). Wu et al. [13] used approximate entropy, fuzzy entropy, and standard statistical functions to detect the envelope of the vibroartrographic signal of the knee. Based on the quoted literature reports, it can be concluded that the dominant segmentation methods of bioacoustics signals are: Hilbert transformation, hidden Markov models, various types of signal entropy, and time-frequency representations. However, in the field of medical image processing, the dominant methods of segmentation take advantage of deep learning and have widely promoted convolutional neural networks (CNN). An and Liu [14] developed a new feedback convolutional neural network algorithm inspired by the feedback mechanism of the human visual cortex. The model first classifies pixel-block samples in the initially extracted image region and then optimises the result by threshold segmentation and morphological methods to achieve the accurate outcome. The proposed method achieved an accuracy of 85.6% in a general tumour image segmentation task, but it also featured a high ability for adaptive segmentation for different medical images. Effectively trained deep learning models require large and representative data sets annotated by specialists. The availability of such databases is often limited as it requires high-level expert knowledge and the manual labelling of images. Girum et al. [15] proposed a new deep learning, weakly supervised model capable of segmenting targets from medical images while generating a labelled data set for deep learning solutions. The method was based on the initial prediction of contours which were then refined by a convolutional neural network that used information from the predicted prior knowledge and the raw input image data. The proposed solution was tested on a prostate clinical target and yielded Dice coefficients of 96.9 ± 0.9% and 96.3 ± 1.3% for computer tomography (CT) scans and echocardiographic images, respectively.

Among different designs of CNNs, U-Net is one of the top fully convolutional network architectures for medical image segmentation tasks [10,16,17]. It extensively uses data augmentation to take advantage of the available labelled samples more effectively. This can be invaluable in the case of small databases containing data which require tedious manual annotating by specialists. The main idea implemented in the U-Net is to refine a usual contracting network with successive layers in which pooling operations are replaced by up-sampling operators with a large number of feature channels. Thus, these layers support higher resolution of the output. The resulting network architecture has no fully connected layers and only uses the valid part of each convolution. Experiments proved the versatility and efficacy of U-Net, as it achieved a warping error of 0.03529%—the best score in the Electron Microscopy (EM) segmentation challenge started at the International Symposium on Biomedical Imaging 2012 (ISBI 2012). It also scored an average intersection over union (IOU) of 92%, which was the highest score in the ISBI cell tracking challenge of 2014 and 2015 [16].

In contrast to 2D data segmentation, one of the most popular neural network architectures in the field of acoustic modelling and speech recognition are recurrent neural networks, mainly long short-term memory (LSTM) models. Zhang and Lu [18] proved that the long short-term memory connectionist temporal classification (LSTM-CTC) architecture outperforms popular deep neural network hidden Markov model (DNN-HMM) algorithms by reducing the word error rate by 1.4% and shortening the model training time by almost 75%. In contrast to many published articles concerning segmentation of heart tones and medical images, no documented techniques of automated segmentation of temporomandibular joint (TMJ) sounds have been found. In a study reported by Djurdjanovic et al. which concerns analysis and classification of sounds generated by the masticatory system, the segmentation of digital acoustic signals was performed manually [1]. However, dentists struggle with the increasing number of patients of different ages who suffer from TMJ disorders [18,19,20]. Therefore, computer-aided solutions would make the TMJ diagnostic process easier and less time-consuming to facilitate dentists in conducting more examinations. It then becomes justified to develop algorithms for extracting episodes of the combined opening and closing of the jaw on the basis of an acoustic signal recorded with an electronic stethoscope. We postulate that it is feasible to design such algorithms, which may in turn facilitate the development of novel TMD automated classifiers to support the diagnosis of the masticatory system. Therefore, in this paper, we implemented and compared two methods of automatic TMJ sound segmentation. The first takes advantage of deterministic digital signal processing. The second, the more complex method, is based on the aforementioned U-Net CNN and LSTM with additional modifications targeting the TMJ use case.

## 2. Materials and Methods

### 2.1. Database

Based on the conducted review of available literature, we found no references which mentioned the existing TMJ sound database. This is why we assume there is a lack of documented open-access repositories of masticatory system acoustic signals. Therefore, in this study, we used our own database created from the sounds recorded with an electronic stethoscope (Littmann Model 3200 Manufacturer 3M Health Care, St. Paul, MN, USA) during a clinical diagnostic trial of TMJ performed in the Dentistry Clinic of Jagiellonian University Medical College in Krakow, Poland (consent of Bioethics Committee number 1072.6120.71.2019). All patients were first diagnosed with standardised official Diagnostic Criteria for Temporomandibular Disorders (DC/TMD) [21,22,23] and further examined by qualified staff. During auscultation, the stethoscope head was applied to the facial skin in the preauricular areas on both sides of the head, and each patient was asked to periodically open and close a jaw with non-standardised frequency, but each recording contained between five and eight repetitions. The sampling rate of the records was 4 kHz and the resolution was 16-bit, which was directly entailed by the stethoscope used. The resulting database consists of 129 recordings collected from 48 patients (30 female and 18 male, with an average age of 34.6) diagnosed with DC/TMD as healthy (29%) or suffering from TMJ disorders (71%). Our designed software tool allowed collected signals to be segmented by a dentist while listening to the recording. This process was performed by labelling the diagnostic sections of the signal, visualised on a personal computer, with a mouse cursor and virtual tags. As a result, all samples from each signal were labelled as ‘1’ when belonging to the segment and ‘0’ when not. The obtained annotated segments with corresponding signal samples were written to the comma separated value (CSV) files which constitute the reference and training set for implemented methods of automated segmentation. The whole dataset contains more than 9.6 million labelled signal samples.

### 2.2. Digital Signal-Processing Method

The first step of the developed segmentation algorithm is the determination of the signal envelope. In principle, there is no single formal definition of a signal envelope, even though the exact mathematical equivalent of this term is an envelope understood as the limitation of a specific family of curves [24]. In practice, however, the concept of an envelope can be treated as an attempt to estimate the amplitude while avoiding over-fitting to the original signal [25]. Meng et al. reported that the calculation of an envelope can be compared to the process of signal demodulation [24]. In our research, however, we adopted the definition of an envelope used by Johnson Jr et al. [26], which recognises it as the instantaneous, low-frequency amplitude changing much slower than the original signal itself. Nonetheless, the word ‘slower’ might be ambiguous when taking into account the non-stationary nature of the analysed signals. First and foremost, the developed segmentation method includes signal preprocessing implemented as the determination of the absolute value of the samples and normalisation. In the next step, the signal is subjected to an elliptical Infinite Impulse Response (IIR) low-pass filter. The filter coefficients are dynamically calculated for each signal because its cut-off frequency is equal to the main harmonic present in the analysed signal, determined by the fast Fourier transform (FFT). The main step of the proposed method of determining the envelope is to use a moving average, i.e., by replacing the samples in a defined time window with the local average calculated in this window. The window width was empirically selected and set to 5000, which gives the equivalent of 1.25 s, taking into account the sampling frequency of 4 kHz [27].

**Figure 2:**
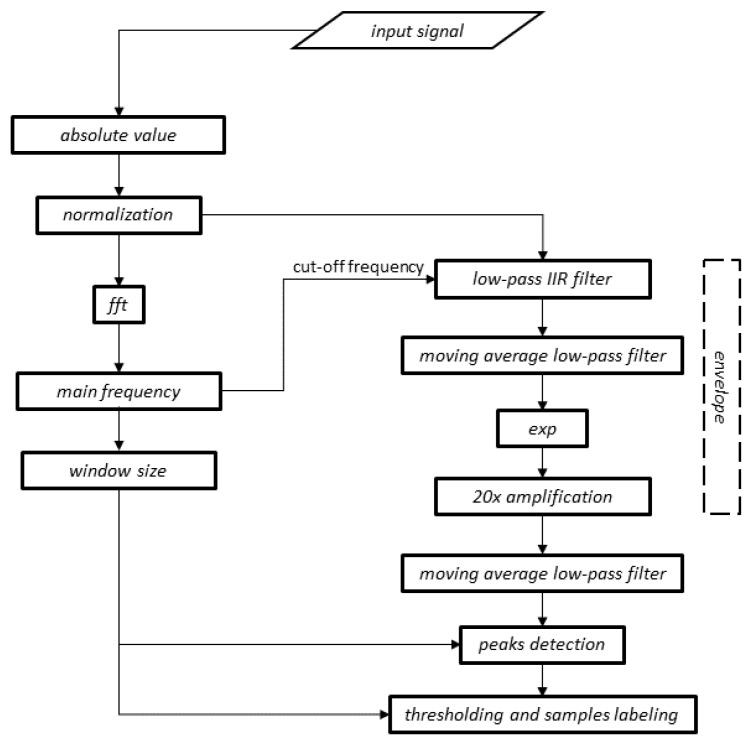
Diagram of the developed method of TMJ acoustic signal segmentation. The cut-off frequency of the low-pass filter is automatically calculated for each signal and is equal to its main frequency.

In order to emphasise the local maxima, the signal subjected to the moving average was then squared, amplified twenty times and filtered again with a low-pass filter of a moving average character in order to eliminate local fluctuations. To localise diagnostic segments, it is necessary to determine the local maxima of the signal obtained in the previous step. This procedure was based on the assumption that the minimum distance between successive peaks is half of the time window, calculated on the basis of the main frequency (the frequency with the highest energy) of the original TMJ acoustic signal. The final step of the algorithm is proper segmentation, which consists of determining the boundaries of the episodes of the masticatory system activity based on the calculated local maxima, assuming that the opening and closing of the mandible is included in 80% of the amplitude of each peak of the envelope. A diagram of the proposed method is presented in Figure 2.

**Figure 3:**
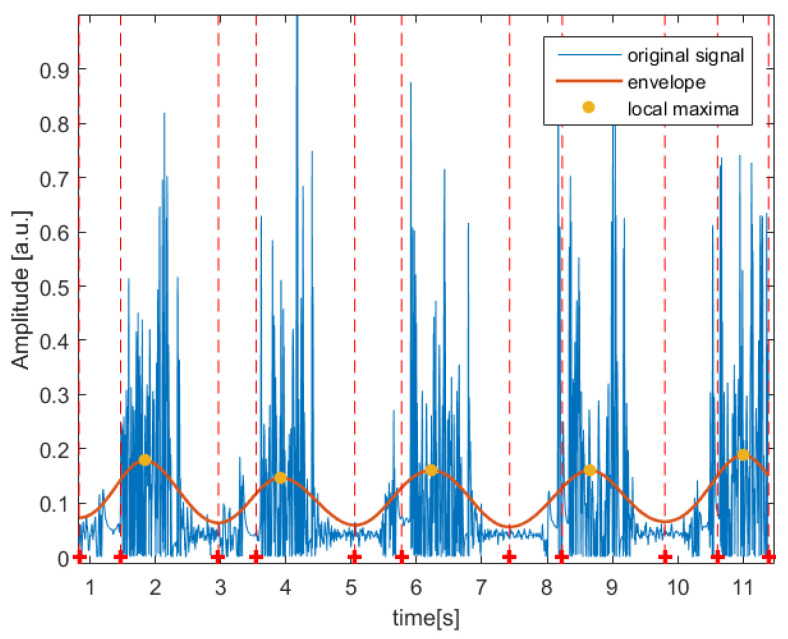
Segmentation result obtained for an example left temporomandibular joint (TMJ) acoustic signal recorded from a patient complaining about pain in left TMJ and diagnosed with left joint arthralgia. The red dashed line corresponds to the beginning and end of every segment.

**Figure 4:**
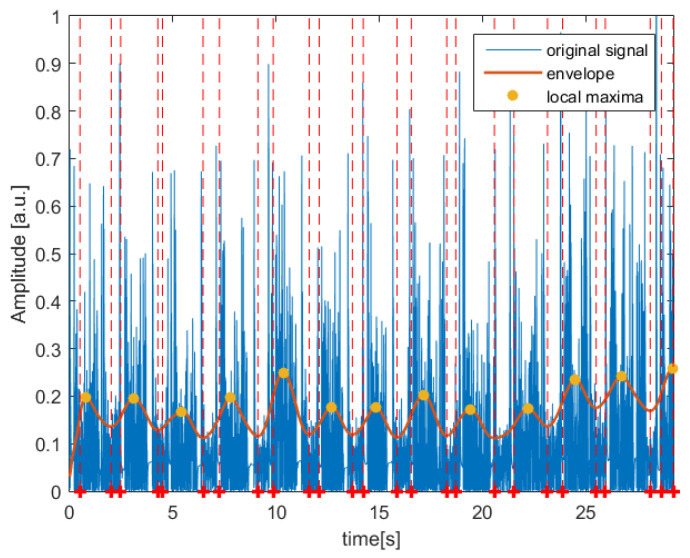
Segmentation result obtained for an example right temporomandibular joint (TMJ) acoustic signal recorded from healthy patient. The red dashed line corresponds to the beginning and end of every segment.

The algorithm was tested on patients who suffer from TMD and also on healthy subjects. In both cases, as presented in Figure 3 and Figure 4, the designed algorithm was able to follow the major frequency present in the signal in order to find the local maxima which constitute the base of each detected segment.

### 2.3. Deep Learning Method

One can find a variety of research in which the authors take advantage of neural networks, especially convolutional and recurrent networks, in the analysis of different kinds of acoustic biomedical signals [28,29,30]. There is no one, unequivocal mathematical formula describing the sophisticated nature of bio-sounds and their propagation in body tissues, nor a detailed model connecting acoustic effects and physical activity of the joint [31]. Hence, neural networks are a perfect tool for TMJ sounds analysis, because during the training process, they attempt to find a non-defined function which maps the input of the network to the output. Such an approach is widely documented in publications related to the heart sound segmentation and classification [29,30,32]. In the case of TMJ acoustic signal segmentation, the result of the deep learning model is a mask which determines if a single signal sample is located in the range of diagnostic segment or not.

Convolutional neural networks consist of convolutional layers which can be considered as a set of filters with parameters (weights) adjusted during training by back-propagation algorithm [33]. A series of such convolutional layers constitute a convolutional network, usually ending with a classifier. In such a setting, the convolutional part can be thought of as a feature-extraction part. For time series, convolutional layers are commonly combined together with recurrent layers, where the first ones act as a preprocessing part and the latter are supposed to analyse the whole time series in order to catch characteristic changes in the preprocessed signal. As stated in the literature [28,29], the most commonly used recurrent layers are LSTMs [34].

Neural network architectures for specific problems are usually designed on the basis of the current state-of-the-art, as there are no formulas for designing architectures. Since 2012, when the AlexNet model was presented [35], many examples of convolutional neural network architectures have appeared designed for some specific tasks, such as classification [36,37], detection [38,39], or segmentation [16]. As our goal is to segment specific parts of TMJ acoustic signals, we decided to start with the current state-of-the-art architecture for segmentation, which is the abovementioned U-Net model [18,19,28].

**Figure 5:**
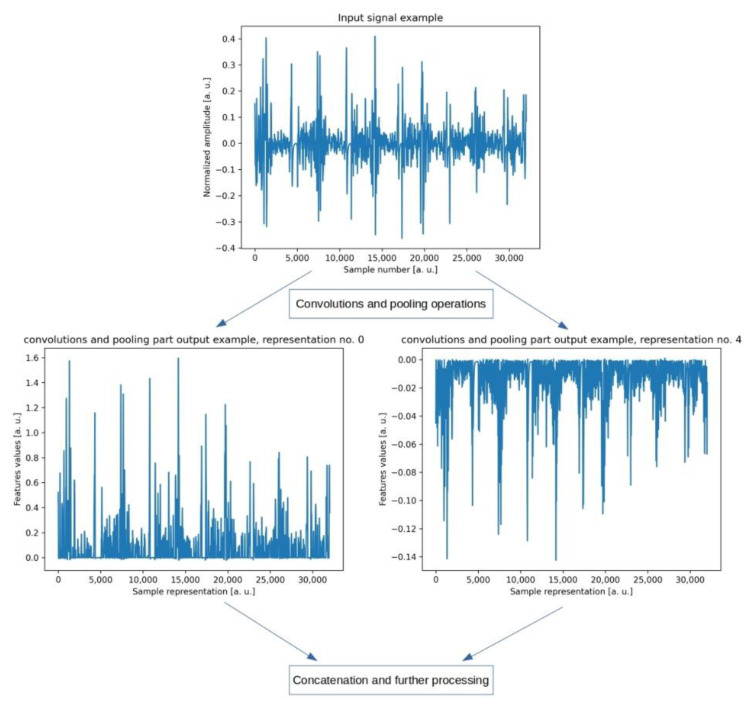
A diagram of the preprocessing step of our model. One can notice that convolutions and pooling operations generate a set of signal representations because each single convolutional layer consists of multiple filters which results in multiple output. Signal representations are shorter than the signal itself because of the pooling layers which discard some processed samples.

One can distinguish two parts of the U-Net’s architecture. The first part consists of convolutions and pooling layers extracting the signal’s local features. Because each convolutional layer is composed of multiple filters, it outputs a set of signal representations. Moreover, after each convolutional layer, there is a pooling layer which discards some of the processed samples, depending on the specific strategy. Setting up a pooling layer after a convolutional layer is a common practice in designing a neural-network architecture [16,35,36,38]. In our case, the first part can be thought of as a preprocessing step, as consecutive layers simply filter the input signal. Eventually, there are multiple signal representations, each of which is shorter than the original signal and contains the most significant features of the signal (Figure 5).

In order to restore the original signal length, the second part of our model extends signal representation to match the original length by placing zeros evenly between existing values and convolving them in order to fill the missing gaps. This operation is possible because of filters for which the parameters are determined during the backpropagation optimisation process. Finally, the output has the same length as the input, but instead of signal amplitude, each value represents belongingness to a class (segment or non-segment).

**Figure 6:**
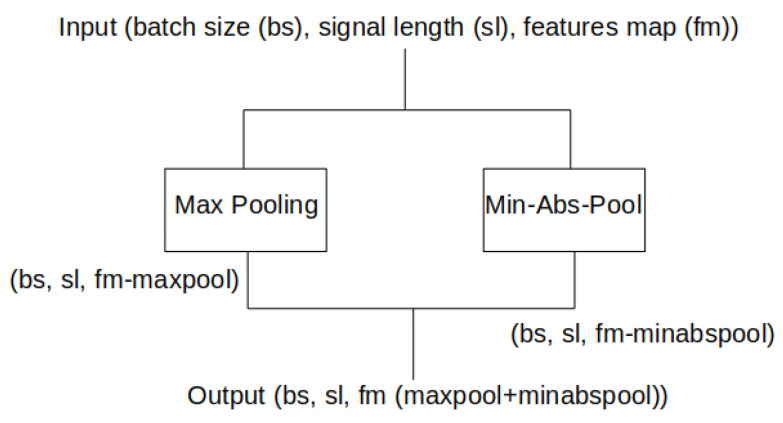
The above image represents a pooling block, which consists of the max pooling layer and the designed min-abs-pooling layer with concatenation of their outputs.

A series of experiments were conducted as far as the network’s architecture is concerned. In the beginning, we started with an original U-Net architecture, which provided no promising results during training because the loss function value was not decreasing. An analysis of feature maps provided us with the conclusion that some important signal features were lost, probably due to max pooling operations after each set of convolutions. For that reason, we designed a min-absolute-pooling layer (Figure 6) which ensures that values close to zero are still propagated to the deeper layers of the network. The min-absolute-pooling section was placed in parallel with the max pooling layer, and outputs from those operations were concatenated in the dimension of channels. Such a modification of the network resulted in a sensitivity increase of around 20%, and 30% in specificity.

The min-absolute-pooling operation can be represented as (1):

(1) P(x)=−M(−|(x)|)

where P is the min-absolute-pooling value, *x* is a signal, and M is max-pooling.

**Figure 7:**
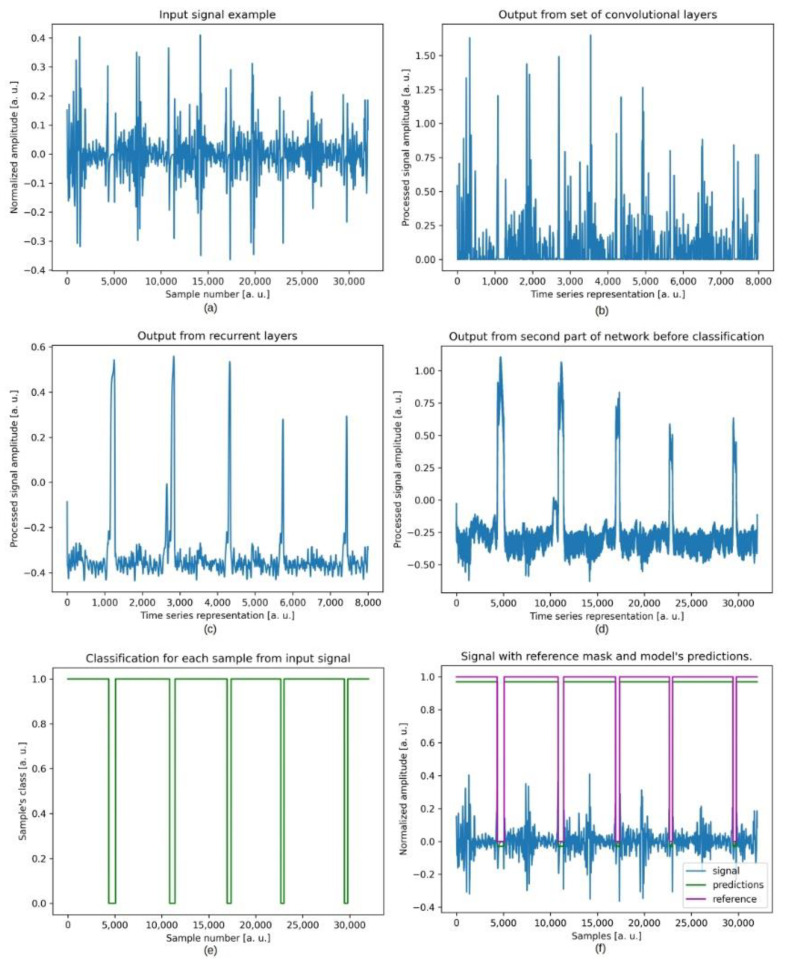
Collection of plots representing output examples from the intermediate steps of our model. (**a**) the original signal (input); (**b**) the signal representation (an arbitrarily chosen set of features) after a set of convolutions and decimation operations; (**c**) output from a set of recurrent layers; (**d**) interpolated output from LSTMs shown in (**c**). The output from (**d**) is then classified if a particular sample belongs to a class which is presented in (**e**); (**f**) original signal together with reference mask and the model’s output mask.

Because acoustic signals are time series in their nature, another modification in U-Net’s architecture was introduced. Instead of simply up-sampling the middle layer, we decided to utilise recurrent layers such as LSTM. Having data in three-dimensional tensor (where the first dimension is a batch size, the second is signal length after pooling, and the last one is the number of features obtained per sample during the calculation of the outputs of consecutive layers) we passed it to an LSTM layer where the time series was the second dimension (signal length) and the features were feature maps. This modification increased the average performance of the resulting model by 5% in sensitivity and 12% in specificity. As a result of numerous experiments, we proposed the final architecture of the model. Figure 7 depicts all intermediate steps of the designed network.

The model consists of two convolutional blocks and three LSTM layers in the middle of the network. Each convolutional block consists of two convolutional layers, max pooling and min-absolute-pooling, which are set up in parallel. The output of each layer is concatenated and then passed to another convolutional block. The second part of the network—the up-sampling block—was left as it is in the original U-Net architecture. In order to justify a design of our model, we decided to visualise outputs from intermediate steps: convolutions and pooling (pre-processing), recurrent analysis with LSTMs, processed signal up-sampling, and the classification of the single input sample.

The original dataset was split into a training set, a validation set and a test set at the very beginning of the experiment. Splitting was done patient-wise, not signal-wise, in order to avoid a situation when signals from the same patient are eventually placed in more than one of the aforementioned sets.

### 2.4. Evaluation

To evaluate the whole test set in the described way, we used the following metrics: sensitivity (2), positive predictivity (3), and Dice coefficient (4). The calculation was performed based on confusion matrix classes such as true positive (TP), true negative (TN), false positive (FP), and false negative (FN).

(2) $$\mathrm{Se}=\frac{\mathrm{TP}}{\mathrm{TP}+\mathrm{FN}}$$

where Se—sensitivity

(3) $$\mathrm{PP}=\frac{\mathrm{TP}}{\mathrm{TP}+\mathrm{FP}}$$

where PP—positive predictivity

(4) $$\mathrm{Dice}=\frac{2*\;y_{t}*y_{p}}{y_{t}+y_{p}}$$

where Dice—Dice coefficient, *y_t_* is a vector of example signal samples’ reference classes, *y_p_* is a vector of the example signal samples’ predictions, * and + operators applied on vectors are element-wise multiplication and addition.

Moreover, specificity (5) and negative predictivity (6) were calculated in order to provide a better evaluation of the results, especially for the negative class (outside of the segment):

(5) $$\mathrm{Sp}=\frac{\mathrm{TN}}{\mathrm{TN}+\mathrm{FP}}$$

where Sp—specificity

(6) $$\mathrm{NP}=\frac{\mathrm{TN}}{\mathrm{TN}+\mathrm{FN}}$$

where NP—negative predictivity

## 3. Results

**Figure 8:**
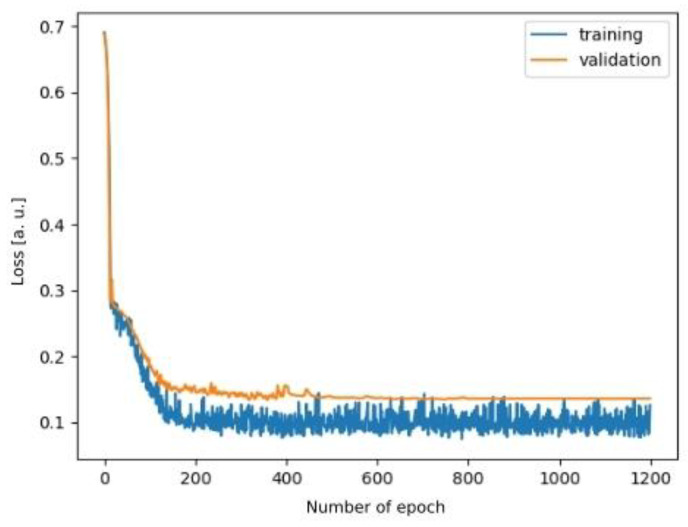
A plot representing the training process (loss function depending on the number of epochs).

The deep neural network model was trained for 1200 epochs with a learning rate of 10^−4^ at the beginning, which was then decreased by a tenth every 400 epochs. The input of the network was defined as 16,000 samples (eight seconds) of the signal because this appeared enough for the model to extract all necessary information (larger input did not provide better results, although the smaller input was preventing the network from learning significant features). As the majority of signals were much longer than eight seconds, we introduced a regularisation technique in order to avoid overfitting by randomly choosing an eight-second-long section of each training example for every epoch. As a result of this approach, the network was not exposed to the same example more than once throughout the whole training process. Such an approach enabled the network to learn features which best described segmented episodes instead of just memorising training examples by heart. Figure 8 captures the training process of the developed model.

The drop of validation error in Figure 8 is only about 7% less than the drop of the training error, which suggests that the proposed model was able to capture the same feature distribution in both sets, even though the whole dataset was relatively small. The fact that the training and validation sets converge proves that the model was not affected by overfitting.

In order to evaluate the model, we needed to supply the eight-second network input with signals of various lengths. To achieve this, we simply split each signal into eight-second intervals and fed them one after another to the network’s input. We then merged each result into the vector of the length of the original signal.

To finish, we calculated the metrics defined in Section 2.4 for DSP and the deep learning method in order to make results for both developed segmentation methods comparable.

As presented in Table 1, the conducted experiments indicate that the proposed methods feature similar Dice coefficients which describe the time precision of the segmentation process. High PP values prove that both algorithms handle the positive class well in comparison to the metrics which evaluate the negative class (Sp, NP). As a result, both methods may be biased towards the positive class representing diagnostic segments of the signal. The reason for this can be the relatively small reference dataset, which possibly promotes positive class samples. A more extensive signal database would certainly enable us to mitigate this limitation.

Nonetheless, the achieved evaluation scores indicate that both of the proposed solutions are potential options in the field of the segmentation of TMJ acoustic signals. The expected expansion of the dataset should improve the results even more.

## 4. Discussion

Despite having been in clinical use since the nineteenth century, the stethoscope still constitutes an indispensable diagnostic tool for most physicians [40]. The simple design of the stethoscope and lack of a need for additional equipment are the advantages of this technique. However, significant knowledge and clinical expertise is required to properly conduct the auscultation. The susceptibility of this method to biased diagnosis, dependent on the physician’s experience, can be resolved through use of electronic stethoscopes and objective digital signal processing. Auscultation is commonly used in cardiology to detect diseases such as heart failure, arrhythmias, valvular insufficiency, left ventricular hypertrophy [41], and pulmonology in cases of sleep apnea and acute respiratory failure [42]. In addition to the well-known examination of the heart, auscultation also supports the diagnosis of other organs and systems; examples include the lungs, intestines [43], and the masticatory system, which is the subject of this paper. In research from 2001, it was found that 12% of United States citizens were affected by temporomandibular joint dysfunction [2], and more recent studies also confirm this thesis. For instance, Gauer and Semidey [6] claim that 10–15% of the population suffer from dysfunctions of the temporomandibular joints or their excessive mobility. However, temporomandibular disorders are difficult to diagnose, and they require a time-consuming clinical examination. Therefore, dedicated signal processing and automated classification solutions should be developed to support dentists on a daily basis. Most signal-processing methodologies require preprocessing and segmentation steps which enable the extraction of segments of the most diagnostic value. This is especially vital in the context of small datasets or, as in the case of TMJ sounds, the lack of reference databases.

Cardiac auscultation and related heart-sound segmentation methods are well established clinical and scientific subjects. Thus, in this paper, we utilised phonocardiography as a reference when developing the presented segmentation methods of TMJ acoustic signals. As it turned out, DSP techniques and LSTM-based models can provide promising results in both fields.

The most significant difference between the proposed deep learning approach and the DSP algorithm is that the first method attempts to mimic the dentist’s perception of TMJ sound diagnostic segments, whilst the second method is theoretical and thus more objective. There is still room for the improvement of both methods. The deep learning architecture may be refined, and the DSP algorithm might be supported by time-frequency signal features. The advantage of the latter is, however, the ease of interpretation, as deep learning models are obscure and nondeterministic in their nature, require an empirical approach, and usually entail a long training process.

## 5. Conclusions

Disorders of the masticatory system are common throughout the population; however, the related diagnosis based on auscultation lacks comprehensive computer-aided support. In this paper, we attempt to fill this niche by presenting two different approaches for the automated segmentation of TMJ acoustic signals. The obtained results suggest that both methods display potential in medical applications, even though the research utilised only a limited dataset. This conclusion confirms the hypothesis that it is possible to develop automated segmentation methods of TMJ acoustic signals.

More acoustic signals collected during clinical TMJ trials would enable the refinement of the proposed algorithms and make them applicable in the development of dedicated TMD classifiers, which in turn may support dentists in the time-consuming TMJ diagnostic process. However, the database created in our research may constitute a reference for further studies and the design of new TMJ sound-processing methodologies.

We hope that our study will contribute to the development of new TMJ sound analysis methods and raise awareness that acoustic diagnosis of the masticatory system is important and should be supported by novel computational methods, as it is done in the case of cardiovascular auscultation.

## Figures and Tables

**Table 1:** Comparison of the evaluation metric calculated for both developed segmentation methods.

Metric	DSP Method	Deep Learning Method
Positive Predictivity (PP)	0.93	0.98
Specificity (Sp)	0.65	0.67
Sensitivity (Se)	0.91	0.98
Negative Predictivity (NP)	0.67	0.74
Dice coefficient (Dice)	0.86	0.85

## Data Availability

The data presented in this study is available on request to the corresponding author.
